# The effectiveness of universal parenting programmes: the CANparent trial

**DOI:** 10.1186/s40359-017-0204-1

**Published:** 2017-10-23

**Authors:** Geoff Lindsay, Vasiliki Totsika

**Affiliations:** 1 Centre for Educational Development, Appraisal and Research (CEDAR), University of Warwick, Coventry, CV4 7AL UK; 2 CEDAR and Centre for Education Studies, University of Warwick, Coventry, UK

## Abstract

**Background:**

There is substantial evidence for the efficacy and effectiveness of targeted parenting programmes but much less evidence regarding universal parenting programmes. The aim of the present study was to evaluate the effectiveness of the CANparent Trial of 12 universal parenting programmes, which were made available to parents of all children aged 0–6 years in three local authorities in England. To the best of our knowledge, this is the first study of universal parenting programmes on this scale.

**Methods:**

Parents accessed a voucher, value £100, to attend an accredited programme of parenting classes. Parents completed measures of their mental well-being, parenting efficacy, parenting satisfaction, and parenting stress, at pre- and post-course. Comparative data were derived from a sample of non-participant parents in 16 local authorities not providing CANparent programmes. A quasi-experimental design was adopted following estimation of propensity scores to balance the two groups on socio-demographic variables.

**Results:**

Following their programme, changes in parenting stress were small and nonsignificant (Cohen’s *d* frequency 0.07; intensity, 0.17). Participating parents showed significantly greater improvements than the comparison group for parenting efficacy (0.89) but not parenting satisfaction (−0.01). Mental well-being improved from 0.29 *SD* below the national norm to the national norm after the course. Parents were overwhelmingly positive about their course (88–94%) but this was lower for improvement in their relationship with their child (74%) and being a better parent (76%).

**Conclusions:**

The CANparent Trial demonstrated that universal parenting programmes can be effective in improving parents’ sense of parenting efficacy and mental well-being when delivered to the full range of parents in community settings. However, there was no evidence of a reduction in levels of parenting stress; nor was there a significant improvement in satisfaction with being a parent. This is the first study of its kind in the UK; although the results point to a population benefit, more research is needed to determine whether benefits can be maintained in the longer term and whether they will translate into better parenting practices.

## Background

A significant challenge to education and public health systems in many countries is the high prevalence of children with behavioural, emotional and social difficulties, with estimates of 10–20% being reported [1–4]. There is now substantial evidence that positive mental health and social development in children is grounded in the quality of parent-child interactions [5, 6] and positive, warm, nurturing environments [7]. Furthermore, positive parenting is associated with reducing the negative impact of social disadvantage [8–12]. In addition to the positive impact on the life chances of individuals, reduction in later negative outcomes will substantially reduce the financial cost to society [13].

Evidence for the effectiveness of parenting programmes to improve parenting skills and reduce child behavioural difficulties is now well established [6, 14–18]. These programmes are typically designed to be targeted at parents with children exhibiting or at risk of developing behavioural, emotional and social difficulties. However, a limitation of this approach is that the programmes can be made available only to a relatively small number of parents who may benefit [19, 20] and also that both recruitment and retention of parents to programmes is often difficult [20]. Consequently, interest has grown in the development of *universal* programmes in addition to *targeted* programmes. The latter have been developed for parents of *indicated* children that have been referred to clinics or *selected* children (high risk children in the community who have not been referred to a clinic). Universal parenting programmes are a public health intervention that could benefit both those in need of parenting support and advice (i.e., those at risk of adverse parenting), but also other families in general, essentially supporting a generation of parents with the expectation that improvements in child well-being will be measurable at the population-level.

Provision of parenting programmes has been a policy feature of the UK government’s Department for Education in England. The government funded local authorities to provide targeted evidence based parenting programmes to parents of children exhibiting or at risk of developing behavioural, emotional and social difficulties through its Parenting Early Intervention Programme (2006–11). Lindsay et al. [21, 22] reported the success of the Parenting Early Intervention Pathfinder (2006–08) using three evidence based parenting programmes in 18 local authorities. On the basis of this evidence, the Parenting Early Intervention Programme was extended across all higher tier local authorities in England (2008–11). Our evaluation of this national roll-out demonstrated that this large scale implementation of targeted evidence based programmes had also been effective [23, 24].

### The CANparent trial

Following the UK general election in 2010, the new Conservative-Liberal Democrat coalition government implemented a change of policy, shifting central government resources away from targeted to universal parenting support. Drivers of this policy change included the desire to provide high quality support to all parents, in order to develop their parenting skills, and a concern that targeting support was potentially stigmatising. The aim, therefore, was to make available high quality *universal* parenting support to all parents of children in their early years, which would enable all parents to access one of a range of quality-assessed parenting programmes.

The Department for Education implemented the two year CANparent Trial in three English local authorities during 2012–14. Although this was not a randomized controlled trial, as described below (Design), we use the term ‘Trial’, where appropriate, as ‘The CANparent Trial’ was the formal designation of the initiative by the Department for Education; otherwise we refer to the ‘study’. Unlike the Parenting Early Intervention Programme, local authorities were not funded to set up and implement parenting programmes. The Department for Education’s aim for the CANparent Trial was to stimulate the supply of parenting programmes suitable for universal use by parents of children 0–6 years, at a cost that would be reasonable to expect at least some parents to pay in a nationwide market of universal parenting support. The Department for Education considered that the supply of good quality evidence based parenting support would potentially be improved by a market driven approach; and that the delivery of such programmes would be normalised by increased supply, as has been the case with antenatal classes in the UK [25], so reducing stigma associated with taking a parenting programme. As a result, it was expected that increased participation by a high proportion of all parents in universal programmes would improve parenting across the country.

It was postulated that development of a market would limit the costs of the study and eventually transfer the main costs from government spending to individuals purchasing their own access to parenting programmes [26]. The overall aim of the study was to examine whether the provision of free parenting programmes in the three CANparent Trial areas would provide sufficient incentive to providers to start offering additional universal programmes nationally, including for parents of children beyond 6 years of age, and whether a universal approach could normalise and de-stigmatise parenting programmes. The Department for Education’s approach was to develop the market in two ways. First, the supply side would be stimulated to attract a number of providers offering different variants of parenting programmes in terms of, for example, content, length and mode of delivery. Quality assurance was achieved by an accreditation process conducted by the Department for Education. The demand side was to be stimulated during the study by provision of a voucher, with a face value of £100, for every eligible parent.

The present paper focuses on the effectiveness of the parenting programmes implemented during the CANparent Trial. This study aims to examine the effectiveness of the parenting programmes with respect to reducing parenting stress and increasing parents’ sense of parenting efficacy, satisfaction with being a parent, and their own mental well-being.

## Methods

### Design

The Department for Education selected three English local authorities in which to implement the CANparent Trial: Camden in London, Middlesbrough in the north east of England, and High Peak in Derbyshire. In a fourth area (Bristol) there were no vouchers; rather, some light touch support was provided. This fourth area is not covered in the present paper. These three local authorities were identified as providing a good mix of locations and demographic spread across England. Sixteen local authorities were selected as a comparison group. Providers of parenting programmes were invited to submit for accreditation to participate in this two year study (April 2012 to March 2014). Fourteen providers were selected by the Department for Education for offering universal parenting programmes appropriate to all parents of children of ages 0–6 years, which met specified quality standards [27].

All parents of children in the relevant age group (both mothers and fathers, male and female carers) in the three areas were eligible to receive a voucher of value £100 to access one of the approved parenting programmes at no cost. Voucher distribution and local support to providers was managed by a delivery consortium funded by the Department for Education. Vouchers were widely available in the three areas through the early years workforce (e.g. children’s centres for pre-five year olds), other community organisations and branches of a national pharmacy; from November 2012 vouchers could also be downloaded from the CANparent website.

The study was designed to produce data that were reported to the Department for Education at points over the two year period. This enabled the Department for Education to learn from the accumulative evidence and make modifications to the Trial as appropriate. Several changes were made, primarily to improve take up by parents in the three implementation areas. For example, in addition to parents resident in the areas, eligibility was given in Year 2 to parents who worked in one of the Trial areas.

To address the question of effectiveness, which is the focus of the present paper, relevant data from the evaluation design are reported, including outcomes from CANparent participants, and comparison data drawn from the comparison local authorities. Initially, the trial design of the CANparent Trial aimed to include evaluation data from 10% of CANparent programme participants, but that was later changed to every participating parent to account for the smaller than anticipated CANparent registration rate.

#### Parenting programmes

At the start of the CANparent Trial, 14 providers offered programmes of parenting classes to eligible parents. Four main delivery models were offered: face-to-face groups; face-to-face one-to-one; blended face-to-face with online and/or self-directed learning (book or CD/DVD); and pure online (Table 1). In order to be eligible for the Trial, providers were required to demonstrate that their programme would meet the Department for Education’s quality standards. These were defined as evidence based principles derived from research into what works to improve parenting skills: specified programme content to include communication and listening, managing relationships (parent/child and parent/parent), play/explore/learning, parenting styles/behaviour, rules and routines, and creating a supportive and nurturing home environment; as well as delivery approach, workforce training and supervision, and evaluation of impact [27: Appendix 4]. Examples of the content of both face-to-face and online programmes included: managing routines and boundaries, supporting each other’s parenting, managing and promoting positive behaviour in the family, understanding the importance of play and exploration, secure relationships, and who’s in charge: What to do when your child says no [27, Appendix 3]. The face-to-face group classes included discussion and role play, with one programme ending with a group meal; these programmes also included support materials and tasks to be carried out between sessions. Programmes differed in length from one of 2 sessions over 2 weeks to others comprising 8, 9 or 10 weekly sessions. Two providers dropped out of the Trial in Year 2 leaving 12 providers (Table 1).

**Table 1:** The CANparent programmes in the three voucher areas

Provider	CANparent class/s	Area/s (Year 1)	Delivery mode (Year 1)	Year 2 changes
Derbyshire County Council	Bringing Up Children	High Peak	• f2f group • f2f 1:1 • online	None
Family Lives	Parents Together	High Peak	• online	Camden also
Family Matters Institute	Triple P	High Peak	• online • blended (3 versions)	Camden & Middlesbrough also
City Lit	[Various names e.g. ‘Once Upon a Time’]	Camden	• blended	None
Coram	Parents as Teachers (Born 2 Learn)	Camden	• f2f group	None
Parent Gym	Parent Gym	Camden	• f2f group • online (live)	None
Barnardos	1–2-3 Magic	Middlesbrough	• f2f group	None
	Caring Start (HighScope)	Middlesbrough	• f2f group	None
	Comfortzone	Middlesbrough	• f2f group	None
	Playgroup Network sessions	Middlesbrough	• f2f group	None
Family Links	The Nurturing Programme – 2-session version	All areas	• f2f group (plus book or DVD)	None
NCT	NCT CANparent	All areas	• f2f • online	Online: Camden & Middlesbrough also; blended option added
Race Equality Foundation	Strengthening Families, Strengthening Communities (SFSC) – adapted version	All areas	• f2f group • online • blended	None
Save the Children	Families and Schools Together (FAST)	All areas	• f2f group	None
Solihull Approach, Heart of England NHS Trust	Solihull Approach Parenting Group	All areas	• f2f group • online	Online: Camden & Middlesbrough also

*Note*: f2f = face-to-face

### Participants

#### Participating parents

Six hundred and seventy five parents participated in the present evaluation study. These are the parents who returned outcome evaluation questionnaires at the start of the study (pre-data). About 30% of participants received a programme of parenting classes in Middlesbrough, 46% in Camden, and 24% in High Peak. The majority (93%) attended a face-to-face group. Approximately half of all parents (53%) were aged between 26 and 35 years, while 26% were aged between 36 and 45 years-old. Fathers comprised just 9% of the group. Seventy four per cent identified as White British, while the largest ethnic minority group were Asians (11%). In terms of education, 18% reported having no educational qualifications, whereas 43% had Level 4 and above, which is equivalent to a university bachelor degree level or higher. Single parents comprised 25% of participants, and 18% of the overall sample lived in the most deprived neighbourhoods of their area [28]. Most parents (41%) had just one child aged 0–16 years, while 36% had two children aged 0–16 years in the household.

Of the 675 parents, 297 did not return post-course outcome data (44% loss to follow up rate). This does not, however, represent a ‘drop out’ rate. Available data on course completion provided by programme providers indicated that 92% parents completed their programme of classes with just 8% identified as non-completers. Comparisons between those who returned post-course data and those who did not indicated no significant differences on parenting stress (Parenting Daily Hassles scale: PDH) [29], or mental well-being (Warwick Edinburgh Mental Well-being Scale: WEMWBS) [30] at pre-course (see Outcome Measures below). In terms of parenting, the differences in Being a Parent (BAP) [31] pre-course scores, both parenting satisfaction and BAP total score were also nonsignificant. However, parents with missing post-data reported higher parenting self-efficacy at pre-course compared to those without missing data (*t* = 2.39, *p* = .017). With respect to demographic characteristics, few differences were present, suggesting non-systematic differences between non-responders and responders: no differences in terms of parental age, gender, area deprivation, marital status, single parent status, number of children in the house, but more people with no/low educational qualifications (*p* = .040) and non-white ethnic background (*p* = .045) had missing data at post.

#### Comparison sample

In the context of the study, 16 local authorities were selected among all English local authorities where CANparent was not operating. These 16 local authorities were nationally representative in terms of key demographics and were selected as comparison areas to the CANparent areas. Using a two-stage random sampling procedure eligible parents (based on Her Majesty’s Revenue & Customs Child Benefit records, which at the time of the study was a non-means tested benefit with a near universal coverage) were identified to create a comparison group to the CANparent Trial. A total of 1535 comparison parents were identified. However, in terms of the effectiveness arm of the Trial (the present study), not all 1535 comparison area parents served as the comparison group, but a randomly selected subset was identified to provide national norms on two measures: the BAP and the PDH. The third outcome measure of the evaluation, WEMWBS, was not completed by comparison parents because national norms were available on this measure [30]. Therefore, among the 1535 comparison parents, a randomly selected sub-sample of 521 parents completed the PDH and another 547 parents completed the BAP. These comparison groups provided norms on the PDH and BAP, against which CANparent scores on these measures were benchmarked.

A further function of the comparison sample was to serve as a comparison group to gauge level of change in the outcomes of the study (i.e., provide a controlled evaluation). For this reason, the comparison group was invited to a repeat administration of the BAP and PDH 8 weeks later, a period that corresponds to the average duration of the parenting programmes. Retention rate for the comparison group was between 34% and 40% (*N* = 209 and 186, for the PDH and BAP, respectively). To enhance the controlled evaluation, we adopted a quasi-experimental design by balancing the two groups across a range of socio-demographic indicators using a propensity score method. Propensity scores are useful for strengthening quasi-experimental designs by balancing the distribution of any pre-intervention differences in the absence of randomisation [32].

### Outcome measures

Three outcome measures were selected to assess important factors that the parenting programmes addressed. Two were selected also because of their successful use in our earlier study of targeted parenting programmes [21–24]. A third measure, of parenting stress, was selected as appropriate for parents of children 0–6 years. In addition, a fourth measure examined parents’ views of the parenting programme they had attended.

#### Being a parent

The Being a Parent scale (BAP) was developed by Johnston and Mash (1989) [31] and comprises 17 items, which are worded positively or negatively, rated on 6-point scales. Johnston & Mash proposed a two factor solution translating into two subscales but Gilmore and Cuskelly (2008) [33] have produced evidence for a three factor solution: Parenting satisfaction (7 items) is an affective dimension reflecting parental motivation, anxiety and frustration with being a parent, for example: ‘A difficult problem in being a parent is in not knowing whether you’re doing a good job or a bad one’. Parenting efficacy (7 items) is an instrumental dimension reflecting the parent’s sense of perceived competence, capability and problem-solving as a parent, for example: ‘Being a parent is manageable and any problems are easily solved’. The third subscale, Parenting interest (3 items) assesses interest in being a parent, for example, ‘Being a good mother/father is reward in itself’. The three scale scores can be aggregated to produce a Total score. Internal consistency in the present study was good, with Cronbach’s alpha coefficients of .80 for Parenting satisfaction, .79 for Parenting efficacy, and .82 for Total score. Comparison group alphas were .82, .74, and .79 for satisfaction, efficacy, and Total score respectively. The internal consistency for Parenting interest was lower at alpha .53 for the CANparent group and .59 for comparison group. Though we included it in the analysis, interpretation of findings of parenting interest should be cautious.

#### Parenting stress

The Parenting Daily Hassles (PDH) [29] is a measure of minor stresses generally experienced by parents in routine interactions with their children and in routine tasks involving children. The PDH comprises 20 items, each of which is rated on a 0–5 scale along two dimensions: frequency of occurrence and intensity (degree of ‘hassle’) as perceived by the parent. Example items include ‘the kids resist or struggle over bedtime with you’, ‘the kids won’t listen - won’t do what they are asked without being nagged’. Internal consistency was very good: Cronbach’s alpha .88 and .92 for intensity in the CANparent and comparison groups respectively; alpha .88 and .87 for frequency in the CANparent and comparison groups respectively.

#### Parent mental well-being

The Warwick-Edinburgh Mental Well-being Scale (WEMWBS) [30] comprises 14 items rated on a 5-point scale. High scores represent greater mental well-being. It measures positive mental health, including subjective experience of happiness and life satisfaction, and perspectives on psychological functioning and personal relationships. Examples include, ‘I’ve been feeling good about myself,’ I’ve been feeling useful,’ and ‘I’ve been dealing with problems well’. It has moderate to high levels of construct validity with nine other comparable scales: median .73, range .42–.77 [30]. It was used successfully in our earlier studies of parenting programmes [21–24]. Internal consistency in the present study was high, alpha .91. The national mean is 51 (inter-quartile range 45–56) [30].

#### How was your class?

A range of perspectives on the programmes taken by the parents was assessed using the How was your class? questionnaire, developed for the present study. The scale comprises eight items rated on a 5-point Likert scale where higher scores represented more positive views. Examples include, ‘I feel more confident as a parent/carer,’ ‘I have learned new parenting skills’ and ‘Overall I was satisfied with my CANparent class.’ [27].

### Procedure

Parents accessed a voucher from an available source and presented this to the programme provider of their choice. Numbers of providers varied between the areas (Table 1). Each voucher had a nominal value of £100 and could be used to access any of the programmes available in their area. Ten providers submitted data on 675 parents who participated in this study.

Upon enrolment with the programme provider, parents provided demographic information. Outcome data at the first session of the course were collected (pre-course) and matched with demographic registration data (*N* = 415). Post-course outcome data were collected again at the end of the parenting programme during the final session, along with course satisfaction data. Most parents (93%) attended a face to face group, and only 7% attended a blended learning group (online and face to face). Parenting programmes could last between 1 and 10 weeks/sessions, but most of them (56%) lasted 6 to 10 weeks/sessions.

### Analysis

To explore the psychological profile of parents who elected to sign up to a universal intervention, we compared CANparent participants’ scores before the parenting programmes with national norms. Comparisons are reported as standardised mean differences (*d*; Table 2) estimated using the mean group difference (prior to the start of CANparent groups) standardised by the pooled standard deviation.

**Table 2:** Comparison of parenting, stress and mental well-being levels *before* the start of the CANparent programmes

	CANparent group		National norms^a^		ES^b^ (95% CIs)
	Mean (SD)	N	Mean (SD)	N	
PDH Frequency	60.53 (10.96)	576	50.58 (11.11)	518	0.90 (.78, 1.03)
PDH Intensity	53.90 (12.56)	501	34.9 (12.26)	515	1.53 (1.39, 1.66)
BAP Satisfaction	25.15 (6.75)	650	28.9 (6.62)	546	−0.56 (−.68, −.44)
BAP Self-efficacy	29.88 (5.74)	648	32.1 (4.70)	547	−0.42 (−.53, −.30)
BAP Interest	14.97 (2.58)	648	15.7 (2.30)	547	−0.30 (−.41, −.18)
BAP Total	70.08 (11.07)	645	76.6 (9.80)	547	−0.62 (−.74, −.50)
WEMWBS	48.39 (8.95)	656	50.7 (8.79)	1749	−0.26 (−.35, −.17)

^a^With the exception of WEMWBS, comparison data came from the comparison group: a randomly selected population sample. WEMWBS comparison data are from the scale’s standardisation sample [30]

^b^ES = effect size; PDH = Parenting Daily Hassles; BAP = Being a Parent; WEMWBS = Warwick Edinburgh Mental Well-being Scale

To address our main research question of CANparent effectiveness, we compared CANparent participants to comparison group parents. A quasi-experimental design was adopted following estimation of a propensity score to balance the two groups on parental age, parent gender, ethnicity, educational qualifications, single parent status, total number of children in the household, Index of Multiple Deprivation, and the Income Deprivation Affecting Children Index [28]. A weight was then created using the reciprocal of the propensity score. This was effective in balancing the two groups in terms of the distribution of most covariates; balance was not achieved for parental age (the comparison groups for BAP and PDH were significantly younger) and single parent status (the comparison group for PDH included 2% additional single parent families). A propensity score weighted multiple regression model was fitted to examine whether group differences were significant for each outcome, controlling for the equivalent baseline measure. A standardised mean difference (*d*) was estimated using the regression coefficient for group [34]. Models were fitted in MPlus 7.4 [35], which allows for a maximum likelihood estimator with robust standard errors to make full use of available data. Maximum likelihood estimation as an approach to dealing with missing data is a good alternative to multiple imputation, and in fact better than maximum imputation when levels of missingness are high [36].

Finally, to explore potential mediators of change within the CANparent group, we plotted change over time (standardised mean difference of the CANparent baseline (pre) to post scores only) against programme characteristics.

## Results

### Parenting and mental well-being of parents who opted to take up a universal offer

CANparent was a universal intervention that was offered to any parent who had a child in the 0–6 age range in the Trial areas. As such, parents could choose whether to take it up or not. To understand the psychological profile of parents who opted to *take up* the intervention, we compared their parenting profile, parental stress and mental well-being (before parenting programme) to national norms. In the case of BAP and PDH, the norms were derived from the randomly selected comparison group of 547 and 521 parents, respectively. WEMWBS norms are available from a standardisation sample of 1749 UK adults [30].

Table 2 includes the effect sizes (*d*) and 95% confidence intervals (CIs) that compared the pre-intervention psychological profile of CANparent participants to national norms. Parenting stress, as measured by the PDH, was substantially greater among the CANparent group than norms on both frequency (*d* = 0.90, 95% CI: 0.78, 1.03) and intensity (*d* = 1.53, 95% CI: 1.39, 1.66) of daily hassles. CANparent participants had a lower level of satisfaction as a parent compared to available norms with a medium effect size (*d* = −0.56, 95% CI: −.68, −.44). Their sense of efficacy as a parent was also lower (*d* = −0.42, 95% CI: −.53, −.30), as was their interest in parenting (*d =* −0.30, 95% CI: -0.41, −0.18) and the BAP total score (*d* = −0.62, 95% CI: −.74, −.50). CANparent participants had lower initial levels of mental well-being (WEMWBS *d* = −0.26, 95% CI: −.35, −.17) compared to UK norms.

These results suggest that parents who opted to take up the universally-offered parenting programmes were experiencing substantially higher levels of parenting stress, had less confidence in their ability to parent, had less satisfaction with being a parent, and a slightly lower level of mental well-being and interest in being a parent.

### Effectiveness of CANparent

We compared parental stress and parenting between CANparent and comparison parents, after propensity weighting. Table 3 presents the weighted means at each time point. Table 4 presents the results of the multiple regression models fitted to examine the effect of group (CANparent vs comparison) on the propensity weighted data, whilst also accounting for the effect of baseline scores at pre-course. A standardised mean difference was also estimated using the standardised group coefficient from the weighted regression model [34].

**Table 3:** Means and standard deviations in the two groups following propensity score weighting (maximum likelihood estimation)

Parenting measure	Group	N	Baseline (pre) weighted mean (SD)	Post weighted mean (SD)
PDH Frequency	CANparent	209	61.86 (9.86)	60.46 (10.72)
	Comparison	211	44.71 (9.24)	47.29 (11.34)
PDH Intensity	CANparent	191	55.37 (11.08)	53.65 (10.53)
	Comparison	213	40.41 (9.44)	42.42 (12.92)
BAP Satisfaction	CANparent	237	24.93 (6.65)	27.13 (5.84)
	Comparison	185	28.21 (6.64)	28.85 (5.99)
BAP Self-efficacy	CANparent	235	29.93 (5.73)	32.12 (4.74)
	Comparison	185	31.66 (4.92)	28.20 (3.03)
BAP Interest	CANparent	235	14.99 (2.57)	15.48 (2.36)
	Comparison	185	11.52 (1.78)	11.89 (1.24)
BAP Total score	CANparent	234	69.84 (10.89)	74.67 (9.62)
	Comparison	186	69.96 (9.68)	67.31 (8.15)

*Note:* ES = effect size; PDH = Parenting Daily Hassles; BAP = Being a Parent

**Table 4:** Propensity weighted multiple regression model results controlling for baseline scores (maximum likelihood estimation with robust standard errors)

Parenting measure	R^2^ (*p* value)	Group beta (SE)	Effect size (95% CIs)
PDH Frequency	53.3% (<.001)	.034 (.041)	.07 (−.12, .26)
PDH Intensity	43.0% (<.001)	.083 (.050)	.17 (−.03, .36)
BAP Satisfaction	43.3% (<.001)	−.003 (.037)	−.01 (−.20, .19)
BAP Self-efficacy	42.7% (<.001)	.402 (.037)	.89 (.68, 1.09)
BAP Interest	55.1% (<.001)	.218 (.045)	.45 (.26, .65)
BAP Total score	49.0% (<.001)	.289 (.034)	.61 (.41, .81)

*Note:* PDH = Parenting Daily Hassles; BAP = Being a Parent

The change in the weighted scores of parenting stress (PDH frequency and PDH intensity) was not significantly associated with group (standardised betas: .034 and .083, for frequency and intensity respectively), and this was also demonstrated by the very small and non-significant effect sizes: PDH frequency *d* = 0.07 (95% CI: -0.12, to 0.26); PDH intensity *d* = 0.17 (95% CI: -0.03, 0.36).

The effectiveness of CANparent was demonstrated through significant gains in parental efficacy, parenting interest and total parenting scores. In particular, the weighted effect size for parenting efficacy demonstrated a large effect in favour of CANparent, *d* = 0.89 (95% CI: 0.68, 1.09); small to moderate gains in terms of parenting interest, *d* = 0.45 (95% CI: 0.26, 0.65), and moderate gains for total parenting scores, *d* = 0.61 (95% CI: 0.41, 0.81). Parenting satisfaction was the only BAP measure not associated with a significant gain for CANparent, *d* = −0.01 (95% CI: -0.20, 0.19).

The WEMWBS was not administered to the comparison group as national norms were available [30]. We compared CANparent WEMWBS scores at post-course (Mean = 51.0, SD: 8.28) with the national norms (Table 2, last row) and this resulted in a near-zero standardised mean difference (effect size *d* = 0.03, 95% CI: -0.08, 0.14). Taken together with the pre-course WEMWBS effect size reported in Table 2, it can be concluded that on average mental well-being of CANparent participants improved from being about one third of a standard deviation below the national norm before they attended the programme to about the national norm after their courses.

### Exploring potential mediators of change

#### Programme characteristics

**Fig. 1:**
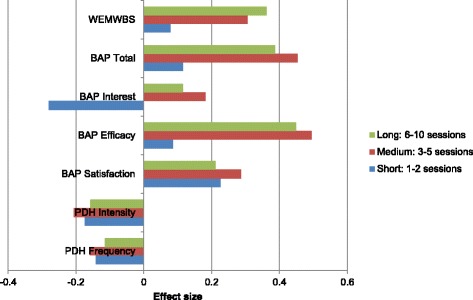
Effect sizes by course length

Type of programme (face to face or blended course) was not associated with any notable differences. Programme length was categorised as short duration (1–2 sessions, *n* = 86 parents), medium duration (3–5 sessions, *n* = 121) and long duration (6–10 sessions, *n* = 258). Short programmes were associated with very little change, on any outcome, other than parenting interest where interest decreased after the programme (*d* = −0.37; 95% CI: -0.62, −0.15; see Fig. 1). Effect sizes for medium duration and long programmes were similar in magnitude. In terms of parental stress, changes were very small regardless of programme length. Some differences were seen in parenting efficacy and Total BAP scores and mental well-being, where medium and longer programmes were associated with small but significant improvements (between a third and half of a standard deviation; see Fig. 1) whereas short programmes were associated with no change (BAP efficacy: *d* = 0.08, 95% CI: -0.12, 0.30 and BAP total: *d* = 0.11, 95% CI: -0.08, 0.31).

### Parents’ satisfaction with the programme

Parents gave consistently positive ratings of their programme across the eight items of the How was your class? scale. The percentage of parents giving negative ratings ranged from just 4% - 5% per item. In comparison, 93% were satisfied or very satisfied with their programme and would recommend a CANparent programme to other parents; 89% said that the programme had met their expectations; 90% had learned new parenting skills; and 88% would like to attend further classes in the future. Most parents felt more confident as a parent (84%), thought their relationship with their child had improved (74%), and that they were a better parent (76%).

## Discussion

In this paper we examined the effectiveness of the CANparent Trial, a universal offer of parenting programmes implemented in three local authorities in England.

### Universality

Recruitment comprised parents in three local authorities who selected to take up the offer of a parenting programme, subsidised by the UK Government. Unlike the earlier roll-out of targeted parenting programmes in England (Parenting Early Intervention Programme), where parents were referred or self-referred for the high levels of (or risk for) behaviour problems in their children, universal recruitment in CANparent was aimed at all parents with a child in the specified age range (0–6 years). This involved ensuring publicity, awareness and availability of the offer in the areas through several means including the early years’ workforce, a national pharmacy and website and then parents deciding on their own whether to sign up or not [27].

The demographic profile of CANparent participants suggested a predominantly White British group where about half of the parents were young (<35 years) and well-educated (degree level and above), and about a quarter were single parents. When we compared the psychological profile of parents who undertook the CANparent universal intervention with national norms, participants in CANparent were experiencing substantially higher levels of parenting stress, replicating a study of universal parenting programmes in Sweden [37] and also were feeling moderately less efficacious and satisfied as parents compared to national norms. They also experienced lower mental well-being, by about a third of a standard deviation.

### Effectiveness

Accounting for any socio-demographic differences in the intervention and comparison groups through a propensity score weight methodology, findings suggested that following parenting programmes there was a substantial improvement in participants’ perception of their efficacy as a parent with a large effect size compared with the comparison group. There was a moderate improvement in parenting interest but not in parenting satisfaction. Interestingly, there was no significant group difference in parenting stress following the intervention.

Overall, these results indicate variable gains for parents receiving universal parenting programmes with significant and substantial improvement in parenting efficacy but no change in parenting stress. Previous evidence on the effectiveness of universal parenting programmes has also been varied. Prinz et al. (2009) [38] reported improvements in child-maltreatment population outcomes of over one standard deviation (Cohen’s *d* range 1.09 to 1.22), effects that remained following a further analysis of their data [39]. Eisner et al. (2012) [40], by contrast, report no consistent effects on a range of 16 relevant dimensions of parenting practices and child behaviour (*d* range 0.00 to 0.24); only two measures had effect sizes reaching 0.2, the lowest level for a ‘small’ effect size. Other studies have also found no effect [41, 42]. Eisner et al. [40] argue that the evidence for positive effects is greatest in studies of universal or prevention parenting programmes for indicated treatment in clinical settings and that small sample size is a key factor as effect sizes decrease in studies with large samples.

With regard to CANparent, the pattern of results suggests that the initiative’s success was in improving parents’ sense of how to be effective as a parent and to gain a sense of improved ability to cope, increasing mental well-being. However, CANparent had limited or no effect on changing parents’ perspectives of their levels of stress associated with, or their satisfaction in, their role as a parent. This is supported by parents’ ratings of the programme, as they were overwhelmingly positive about the experience, but they were less likely to report a better relationship with their child or that they were a better parent.

In part this is likely to depend on the ‘dosage’, which we were able to assess given the different lengths of programmes in CANparent. We found that short courses were associated with very limited changes on the parenting satisfaction and well-being measures, and there was even a negative effect of short courses on parents’ interest in parenting. Longer courses, by contrast, were more effective in improving parents’ self-efficacy as a parent and their mental well-being but there was no difference between programmes of medium length (3–5 sessions) compared with longer courses (6–10 sessions).

Targeted parenting programmes are typically longer than those in CANparent [43] but are intended to improve parenting skills which are substantially less than optimal. Whereas some universal programmes also address this subgroup [44], CANparent was designed to address the full demographic range of parents, who generally have a higher level of positive parenting skills. Nevertheless, the finding of a plateau effect for course length is potentially important with implications for cost effectiveness. Interestingly, we found no differences for fully online and blended courses (i.e., those that offered remote and face to face contact). Comparative studies of specific parenting programmes are rare but Lindsay et al. (2011) found no relationship between targeted programmes and course length for Triple P, Incredible Years, and Strengthening Families Strengthening Communities [22], and for these programmes plus the Strengthening Families 10–14 programme in a second study [24]. Subject to further replication, this finding in the present study has implications for cost effectiveness considerations in future larger roll outs of universal interventions.

These findings indicate that a universal level intervention such as CANparent can have measurable benefits on parents’ self-efficacy and mental well-being, though parenting stress does not appear to reduce substantially. While some programme characteristics may mediate the effectiveness, it is unclear at this stage whether gains can be maintained over a longer period or translate into actual improvements in parenting practice. Future research needs to include follow up evaluation, along with measures of parenting practices.

### Limitations

It was not practical within the Department for Education’s design of CANparent to devise an RCT which was both practical and ethical. Comparative data were derived from a sample of non-participant parents in local authorities not providing CANparent programmes: A quasi-experimental design was adopted following estimation of propensity scores to balance the CANparent (participant) and comparison (non-participant) groups on socio-economic variables. Although not an RCT this was a strong, ethically acceptable design for this study. A limitation is that, as there was no randomization to the intervention and comparison groups, it was not possible to control for the level of motivation to change in the two groups. Given the relatively small numbers of parents that enrolled with some programmes, it was not possible to compare the possible differential effects of the different programmes within the study, although we were able to demonstrate that effectiveness was related to the length of the programme whereas there was no difference between the mode of delivery (face-to-face; or blended face-to-face and online). This design was a function of the deliberate aims of the UK Government’s Department for Education that CANparent should be a study of a number of different universal parenting programmes that wished to take part and met the Department for Education’s quality criteria, in order to examine the development of a market of providers. A second limitation in the set-up of the CANparent Trial by the Department for Education, that registration and parent demographic information be recorded by a separate organisation, resulted in a reduction in the overall number of participants for whom available programme registration (including completion) and demographic characteristics could be matched to their evaluation data. Third, although the PDH includes items that relate directly to child behaviour, it focuses on parents and is not primarily a means to measure child behaviour. Measures of parental reported child behaviour are useful in assessing parenting programme effectiveness as, although the main target of the programme itself is parent change, the ultimate intention is also to address child behaviour.

## Conclusions

The CANparent Trial was designed as a universal intervention on the basis that all parents will benefit from support to develop their parenting skills and that, as a consequence, this public health approach would reduce the prevalence of child behavioural difficulties as they develop. A large positive effect of the CANparent Trial was found with respect to parents’ sense of efficacy with being a parent, and a small effect on their parenting interest and their mental well-being. There was no evidence of a reduction in levels of parenting stress or their satisfaction with being a parent as a result of the programmes.

This was a unique study in the UK of a universal approach to the provision of parenting programmes. Our results indicate that universal parenting programmes may have a positive effect on parents’ increase in their sense of their efficacy as a parent and on their mental well-being when delivered to the full range of parents in a community. In the absence of data on actual parenting practices and child behaviour problems, we cannot yet determine whether universal interventions have measurable benefits for overall levels of behaviour problems in the population.

## Abbreviations

BAP: Being a Parent
PDH: Parenting Daily Hassles
WEMWBS: Warwick Edinburgh Mental Well-being Scale
